# Treatment of emphysematous polycystic renal infection in patients with autosomal dominant polycystic kidney disease: Feasibility and limitations of percutaneous cyst drainage 

**DOI:** 10.5414/CNCS111450

**Published:** 2024-12-20

**Authors:** Hisashi Sugimoto, Tatsuya Suwabe, Shigekazu Kurihara, Yuki Oba, Daisuke Ikuma, Hiroki Mizuno, Akinari Sekine, Masayuki Yamanouchi, Katsuyuki Miki, Takayoshi Yokoyama, Yuki Nakamura, Yasuo Ishii, Takehiko Wada, Naoki Sawa, Yoshifumi Ubara

**Affiliations:** Nephrology Center and the Okinaka Memorial Institute for Medical Research, Toranomon Hospital, Tokyo, Japan

**Keywords:** emphysematous polycystic renal infection (EPRI), percutaneous cyst drainage, autosomal dominant polycystic kidney disease, surgical nephrectomy

## Abstract

Emphysematous polycystic renal infection (EPRI) has a poor prognosis with conservative management, and early surgical nephrectomy has been recommended. However, percutaneous cyst drainage may be a possible treatment option. We experienced 6 patients with autosomal dominant polycystic kidney disease (ADPKD) presenting with EPRI. Three patients developed EPRI after renal transarterial embolization (TAE), and the other 3 developed EPRI independently of renal TAE. Two of the patients had only one cyst with gas formation, and the causative organism was sensitive to antibiotics; these patients were cured by cyst drainage and antibiotic therapy. However, in 3 patients with severe renal enlargement and gas formation in multiple cysts, the causative organism was antibiotic resistant and cyst drainage was not effective, so surgical nephrectomy was performed and the disease effectively treated. Surgical nephrectomy should be considered in patients with multiple cysts with gas formation and severe renal enlargement.

## Introduction 

Emphysematous pyelonephritis (EPN) is a severe renal infection with gas (air) formation and necrotic lesions in the renal parenchyma and perirenal tissue; it is particularly common in diabetics and post-renal transplant patients receiving immunosuppressive treatment, especially women [1]. In patients with autosomal dominant polycystic kidney disease (ADPKD), intrarenal infection with gas (air) production has also been reported as a severe form of cyst infection and is termed emphysematous polycystic renal infection (EPRI). EPRI has been reported mainly in patients on dialysis and those with end-stage renal failure and renal transplant. The characteristics of EPRI remain unclear because of the paucity of reports, although one article emphasized the need for prompt nephrectomy [2]. 

We experienced 6 patients with EPRI. Here we report on the limitations of percutaneous cyst drainage and when to proceed to surgical nephrectomy. 

## Case 1 

A 77-year-old woman with a history of ADPKD and diabetes mellitus was admitted to hospital for examination of urinary tract infection, appetite loss, fatigue, and hypotension. For 5 years, she had experienced frequent urinary tract infections, each of which could have been treated with levofloxacin (new quinolone synthetic antibacterial agents). Levofloxacin was also administered for the current episode of urinary tract infection, but the urinary tract infection did not improve and the patient was admitted to our hospital. 

On admission, the patient was 163 cm tall and weighed 59.5 kg. Her blood pressure was 96/61 mmHg; heart rate, 71 beats per minute and regular; oxygen saturation (SpO_2_), 97% on room air; and temperature, 36.3 °C. Heart and breath sounds were normal. Edema was present in the lower extremities. 

Her level of consciousness was reduced, as shown by a Glasgow Coma Scale score of E3-V4-M5 and Japan Coma Scale (JCS) score of II-10. She reported no abdominal pain, and no abdominal tenderness or costovertebral angle tenderness were evident. 

**Figure 1:**
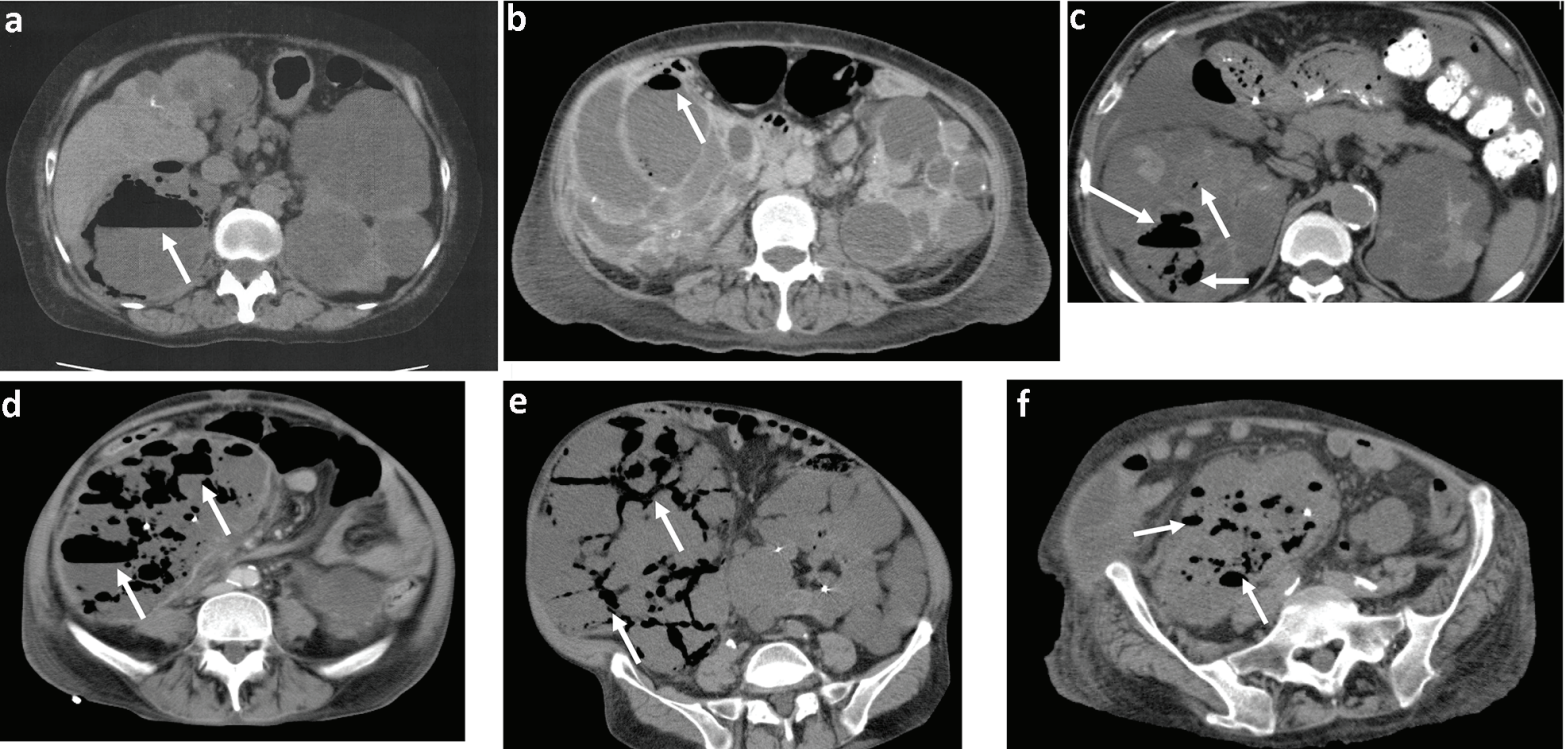
Computed tomography images. a: Case 1: Horizontal surface border structure between fluid and gas (arrow) was seen only in one 10-cm cyst in the upper pole of the right kidney. b: Case 2: Significant gas formation (arrow) was found in only one 5 × 6 cm large cyst in the right kidney. c: Case 3: More than three cysts with gas formation were seen in the right kidney. d: Case 4: Gas formation as observed in multiple cysts of the right kidney. e: Case 5: Gas formation was seen in numerous cysts of the right kidney. f: Case 6: Gas formation was seen in numerous cysts of both kidneys.

Laboratory findings were as follows: leucocytes, 9,800/μL; serum creatinine, 1.5 mg/dL; estimated glomerular filtration rate (eGFR), 26.3 mL/min/1.73m^2^; and C-reactive protein (CRP), 16.8 mg/dL. The urinary sediment contained more than 50 leucocytes per high-power field. A computed tomography (CT) scan showed numerous cysts in both kidneys, and horizontal surface border structure between fluid and gas (niveau formation) in one 10-cm cyst in the upper pole of the right kidney (Figure 1a). The patient had a high CRP level, marked loss of appetite, and low blood pressure, and sepsis was suspected. Because EPRI was diagnosed by CT, the patient underwent cyst drainage under ultrasonic guidance according to a previously described method [3], followed by repeated washing with fresh saline solution until the cyst effluent normalized, and then filling of the cyst with minocycline 2 g. The drainage, washing, and antibiotic administration were repeated for 7 days. 

The first cyst puncture sample was brownish pus, and *Klebsiella pneumoniae* was detected in bacterial culture; however, no causative bacteria were detected in blood or urine samples. A levofloxacin susceptibility test for *Klebsiella pneumoniae* was judged sensitive. According to the sensitivity report, *Klebsiella pneumoniae* was sensitive to levofloxacin and was used in the above case but was not effective. It has been suggested that EPRIs may not even respond to administration of sensitive antibiotics. Therefore, drainage was proposed as the next treatment option. After the 7 days of cyst drainage, washing, and minocycline treatment, the patient was treated with the following antibiotics to which *Klebsiella pneumoniae* is sensitive: intravenous meropenem 0.5 g/day for 7 days, cefmetazole 1 g/day for 15 days, cefepime 0.5 g/day for 26 days, and cefotaxime 0.5 g/day for 10 days. Serum CRP still remained elevated (3.77 mg/dL). Serum CRP did not decrease to below 0.3 mg/dL and complete cure of the infection was not achieved. Therefore, on day 22 of hospitalization a second cyst drainage was performed with the aforementioned regimen for 1 week, and a third one was performed on day 28. 

**Figure 2:**
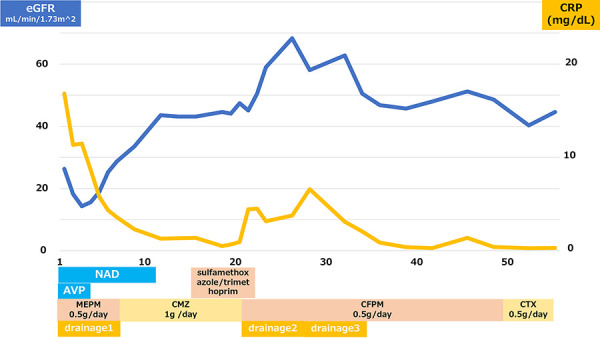
This describes the treatment course, renal function, and CRP trends in Case 1. The patient’s overall condition improved after multiple drainages.

It took 59 days from admission to discharge before the CRP improved (0.30 mg/dL) and the cyst infection was completely cured (Figure 2). During hospitalization, the patient’s blood pressure decreased because she developed sepsis during first cyst drainage, so norepinephrine and vasopressin were administered. After the sepsis treatment started to take effect, blood pressure normalized. 

## Case 2 

A 56-year-old woman was admitted to hospital with complaints of right-sided abdominal pain and fever. She had been diagnosed with schizophrenia at the age of 16 and had started hemodialysis because of ADPKD at the age of 54. Two weeks prior to this admission, she presented with a decreased level of consciousness, fever of 37.5 °C, leukocytosis (10,600/µL), and elevated CRP (19.5 mg/dL). Treatment with minocycline was initiated. However, the patient’s symptoms worsened despite treatment, so she was transferred to our hospital. 

On admission, the patient was 155 cm tall and weighed 58.2 kg. Her blood pressure was 116/59 mmHg; heart rate, 105 beats per minute and regular; SpO_2_, 94% on room air; and temperature, 37.2 °C. Heart and breath sounds were normal. Her JCS score was II-20. She reported no pain, and no abdominal tenderness or costovertebral angle tenderness was evident. Edema was not present in the lower extremities. Laboratory findings were as follows: leucocytes, 11,800/μL; serum creatinine, 3.6 mg/dL; eGFR, 11.2 mL/min/1.73m^2^; and CRP, 11.8 mg/dL. A CT scan showed numerous cysts in both kidneys. Gas formation was seen in only one 5 × 6 cm large cyst in the right kidney (Figure 1b). Diffusion-weighted magnetic resonance imaging (MRI) showed one hyperintense cyst that contained gas and also three hyperintense cysts without gas (two in the right kidney and one in the left kidney). EPRI was diagnosed. The cyst with gas and also the three without gas that were hyperintense on MRI were drained and treated in the same way as in case 1. The antibiotic tazobactam/piperacillin combination 2.25 g/dose was administered 3 times a day (i.e., every 8 hours). Four puncture specimens were positive for *Escherichia coli*, which was reported to be sensitive to this antibiotic. The treatment was effective, and the patient was discharged 21 days after admission. However, 2 years later, she again developed EPRI in the right kidney in a different cyst than the previous one. Surgical nephrectomy was immediately performed, and the infection resolved. 

## Case 3 

A 64-year-old man with ADPKD who had been on continuous ambulatory peritoneal dialysis (CAPD) for 3 years was admitted to another hospital with a fever of 38.9 °C and CRP of 24.6 mg/dL. He was treated with ampicillin-sulbactam, meropenem, ciprofloxacin, and vancomycin but was transferred to our hospital after not responding to treatment. A CT scan revealed more than three cysts with gas formation in the right kidney, and EPRI was diagnosed (Figure 1c). The cysts were drained, and multidrug-sensitive *Klebsiella pneumoniae* was detected in the effluent of all three. After drainage, the patient’s condition did not improve, so surgical resection of the right kidney was performed 3 days later; the patient subsequently recovered. 

## Case 4 

A 52-year-old man with ADPKD who had undergone CAPD for 4 years followed by hemodialysis for 3 years was admitted to our hospital with fever and enlarged kidneys. After 1 month of antibiotic treatment with ciprofloxacin, the fever resolved, and the enlarged kidneys were treated by bilateral transarterial embolization (TAE) according to previous reports [4, 5, 6]. A fever of 39 °C developed after TAE and then resolved; however, it returned 1 week later. A CT scan showed gas formation in multiple cysts of the right kidney, and EPRI was diagnosed (Figure 1d). Surgical nephrectomy of the right kidney was performed because multidrug-resistant *Escherichia coli* was detected during cyst drainage. The fever resolved 2 weeks later [7]. 

## Case 5 

A 74-year-old man with ADPKD who had been on dialysis for 1 year was admitted for TAE for renal enlargement. After TAE, he developed prolonged fever, and a CT scan showed gas formation in numerous cysts in the right kidney and a few cysts in the left kidney (Figure 1e). EPRI was diagnosed. Because it was considered difficult to drain all the cysts, the right kidney was removed 7 days after TAE. However, because of prolonged fever, the left kidney was also removed 2 weeks later. Subsequently, the patient’s fever resolved. 

## Case 6 

A 68-year-old woman with ADPKD and a 6-year history of hemodialysis was admitted for bilateral TAE for renal enlargement. 16 days after TAE, the patient developed a fever, and a CT scan was performed. Gas formation was seen in numerous cysts in both kidneys, and EPRI was diagnosed (Figure 1f). The patient refused surgery, so cyst drainage was repeated three times; however, the patient died 2 months later. Multidrug-resistant *Escherichia coli* was initially detected in the cyst effluent, followed by *Candida albicans.*

## Discussion 

Here, we presented 6 patients with ADPKD presenting with EPRI. Two patients had only one cyst with gas formation (cases 1 and 2) and were successfully treated with cyst drainage (Table 1). One patient on CAPD had numerous cysts with gas formation (case 3), and surgical nephrectomy was performed because it was considered difficult to treat the cysts by cyst drainage alone; the patient subsequently improved. In 2 of the patients (cases 4 and 5), surgical nephrectomy was performed for severe renal enlargement and numerous cysts with gas formation that were considered difficult to treat by drainage alone; the patients improved after the operation. One patient (case 6) with the same condition as the previous 2 patients was told that she needed surgery, but she refused it, so cyst drainage was performed; however, the patient subsequently died. 

TAE is a common procedure at our hospital, and since 1996 we have performed TAE for enlarged kidneys in many patients: as of December 2023, 1,400 patients have undergone renal TAE at our hospital, and 3 of these patients also had EPRI (cases 4, 5, and 6). 

To put our cases into perspective, we performed a literature search for previously reported cases of EPRI and found six reports [2, 8, 9, 10, 11, 12], as well as a report on EPRI after arterial embolization in a patient with severe renal enlargement due to ADPKD [7]. We briefly discuss these reports below. 

Erkoc et al. [8] reported the case of a 40-year-old male patient with ADPKD on hemodialysis who was diagnosed with EPRI because of the presence of gas in a cyst in the right kidney. Treatment was started with antibiotics only but was ineffective, and the patient died of sepsis. The authors reported the need for prompt nephrectomy in patients with EPRI. 

Sooraj et al. [9] reported on a 57-year-old man with end-stage renal disease (serum creatinine, 8.0 mg/dL) before dialysis who was diagnosed with EPRI because of gas formation in one of the cysts in the right kidney. The patient was treated with high-dose antibiotics, started on hemodialysis, and underwent emergency nephrectomy, but the postoperative course was poor and the patient died. The causative organism was identified as *Escherichia Coli*. The authors concluded that in such cases it is important not to operate too late. 

Kim et al. [10] reported the case of a 62-year-old woman on hemodialysis who was diagnosed with EPRI but had gas formation only in a cyst in the left kidney. The patient was cured by antibiotics and cyst drainage. Although *Escherichia coli* was detected in cyst effluent culture, no bacteria were detected in blood or urine samples. Because the patient had a history of cystitis and pyelonephritis, the disease was assumed to have developed from a urinary tract infection. 

Nagaraju et al. [11] reported the case of a 61-year-old man undergoing CAPD who was found to have gas formation only in the left kidney and was diagnosed with EPRI. The causative organism was *Klebsiella pneumoniae*, and the patient went into remission with antibiotic therapy alone. 

Watanabe et al. [2] reported on a 44-year-old woman diagnosed with EPRI because of gas formation in multiple cysts in the right kidney. This patient’s serum creatine level had risen to 17.0 mg/dL when she visited the hospital, so hemodialysis was performed immediately, followed by antibiotics and CT-guided cyst drainage three times; subsequently, nephrectomy was performed, and the patient was cured. The causative organism could not be identified. 

Zijl et al. [12] reported a case of a renal transplant recipient with ADPKD who was diagnosed with EPRI because of the presence of gas formation in a cyst in the right native kidney. The patient was cured by nephrectomy. The causative organism was *Clostridium perfringens*. 

Taken together, these articles suggest that antibiotic therapy alone may be curative for EPRI, but that the disease is often resistant to treatment. Renal cyst drainage is the next treatment option, and surgical nephrectomy is the final treatment option if antibiotics and cyst drainage are ineffective. It should be noted that surgical nephrectomy has a poor prognosis if performed at a late stage [2]. 

On the basis of our six cases and the above, we conclude that patients with a single cyst with gas formation can be treated by cyst drainage alone but that those with multiple cysts with gas formation and severe renal enlargement are difficult to treat by drainage alone and require surgical nephrectomy. 

## Statement of ethics 

This investigation was performed in accordance with the Declaration of Helsinki. The patients gave their written informed consent for this case series to be published. 

## Authors’ contributions 

Hisashi Sugimoto: first author. Tatsuya Suwabe, Shigekazu Kurihara, Yuki Oba, Daisuke Ikuma, Hiroki Mizuno, Akinari Sekine, Masayuki Yamanouchi, Katsuyuki Miki, Takayoshi Yokoyama, Yuki Nakamura, Yasuo Ishii, Takehiko Wada, Naoki Sawa: literature research. Yoshifumi Ubara: last author. 

## Funding 

No funding was received.

## Conflict of interest 

The authors have no competing financial interests or conflicts of interest to declare. 

**Table 1.** The table summarizes clinical information for six cases.

**Case**	**1**	**2**	**3**	**4**	**5**	**6**
Age (years)	77	56	64	52	74	68
Sex	Woman	Woman	Man	Man	Man	Woman
Years on dialysis	CKD stage4 (eGFR 26.3)	CKD stage 4 (eGFR 11.2)	CAPD 3 years	CAPD4 years + HD 3 years	HD 1 year	HD 6 years
Triggers	Preceding cystitis	Unknown	Unknown	After renal TAE	After renal TAE	After renal TAE
Air formation (right kidney)	One cyst	One cyst	At least 3 cysts	Many cyst	Many cyst	Many cysts
Air formation (left kidney)	None	None	None	None	Small cyst	Many cysts
Causative agent	Multidrug-sensitive *Klebsiella pneumoniae*	Multidrug-sensitive *E. coli*	Multidrug-sensitive *Klebsiella pneumoniae*	Multidrug-resistant *E. coli*	Not inspected	Multidrug-resistant *E. coli* and *Candida Albicane*
Cyst drainage	Yes (three times)	Yes (1 time)	Yes (1 time)	Yes (1 time)	No	Yes (3 times)
Surgical nephrectomy	None	None	Right nephrectomy	Right nephrectomy	Both kidneys	No
Prognosis	Survive	Survive	Survive	Survive	Survive	Death
History of diabetes	Yes	Yes	No	No	No	No
History of urinary tract infection	Yes	Yes	No	No	No	No
